# Factors influencing Lyme borreliosis risk perception in Europe: a cross-sectional multi-country survey study

**DOI:** 10.1186/s12889-025-23722-z

**Published:** 2025-08-01

**Authors:** Emily Colby, L. Hannah Gould, Ye Tan, Andreas Pilz, Gordon Brestrich, Jennifer C. Moisi, James H. Stark

**Affiliations:** 1 Global Vaccines and Anti-Infectives Medical Affairs, Pfizer, Pfizer US Commercial Division, New York, NY United States; 2 Evidence Generation Statistics, Pfizer Research and Development, Cambridge, MA USA; 3 Global Vaccines and Anti-Infectives Medical Affairs, Pfizer Corporation Austria, Vienna, Austria; 4 Global Vaccines and Anti-Infectives Medical Affairs, Pfizer Pharma GmbH, Berlin, Germany; 5 Global Vaccines and Anti-Infectives Medical Affairs, Pfizer US Commercial Division, Paris, France; 6 Global Vaccines and Anti-Infectives Medical Affairs, Pfizer Cambridge, Cambridge, MA USA

**Keywords:** Lyme borreliosis, Ticks, Risk perception, Europe, Survey research

## Abstract

**Background:**

Lyme borreliosis (LB) is the most common vector-borne disease in Europe. Research on factors that shape LB risk perception in Europe is limited. This study explores the potential drivers of LB risk perception in the general adult (18–65 years) population of twenty European countries, such as urbanicity, socioeconomic status, dog ownership, gender, age, and tick and LB diagnosis history.

**Methods:**

Data were obtained from a 2022 survey of 28,034 adults aged 18–65 years in twenty European countries. The survey included questions on knowledge, attitudes, and practices related to ticks and LB. Respondents were categorized into three income levels (low, middle, or high) based on country-specific income tertiles. Descriptive statistics were calculated, and differences in responses to survey items by income level were assessed using chi-square tests. Risk perception was measured from a survey question asking respondents to rate their risk of contracting LB on a five-point scale from “very low risk” to “very high risk.” Ordinal logistic regression modeled the relationship between predictor variables and LB risk perception.

**Results:**

Having a past LB diagnosis (adjusted odds ratio [AOR]: 3.96, 95% confidence interval [CI]: 3.38–4.64) was most strongly tied to increased LB risk perception. Knowing someone with a past LB diagnosis or having a past tick bite, were also significantly associated with higher risk perception (AOR: 2.11, 95% CI: 1.97–2.27, and AOR: 1.71, 95% CI: 1.60–1.84, respectively). High-income respondents were most likely to report a past tick bite (55.4%, 95% CI: 53.5–57.3%) and a past LB diagnosis (13.5%, 95% CI: 12.1–14.8%).

**Conclusions:**

Experience with ticks and LB (i.e., via bites or diagnoses) may play a key role in shaping LB risk perception among European adults. Dog ownership and demographic factors such as gender and age may also influence LB risk perception. These results could help LB educational campaigns addressing knowledge and perception gaps.

**Supplementary Information:**

The online version contains supplementary material available at 10.1186/s12889-025-23722-z.

## Background

Lyme borreliosis (LB) is the most common tick-borne disease in Europe [1], with an estimated 129,000 cases reported each year from the twenty-five European countries with LB public health surveillance systems in place [2]. Incidence and risk areas for LB are expanding throughout Europe, making it an important public health concern [3]. The bacteria in the *Borrelia burgdorferi* sensu latu complex causes LB and is transmitted to humans through the bite of infected *Ixodes* spp. ticks. LB often presents as a localized disease in the form of an erythema migrans (EM) rash, which is typically treatable with antibiotics. However, if left untreated, the bacteria can disseminate to other organ systems, leading to severe forms of disease, such as Lyme neuroborreliosis or Lyme carditis [4].

Human risk for LB infection is multifactorial, requiring the presence of an environment that is hospitable for infected ticks coupled with human behaviors (e.g., outdoor activities) that put people in contact with those environments. The former is predicated on geographic factors that produce favorable tick habitats (e.g., warmer climates, landscape fragmentation, etc.) [5], while the latter is dependent on access to tick-conducive environments, such as green spaces, and the use of preventive behaviors like conducting tick checks and using insect repellents that help mitigate risk. Effective prevention and control measures thus depend not only on medical intervention, but also on public awareness and the adoption of preventive behaviors, such as wearing protective clothing, to reduce exposure to infected ticks. Individual uptake of these preventive measures is at least in part a function of perceived susceptibility to LB.

Prior studies in various settings have described knowledgeability and regional endemicity as important factors shaping LB risk perception [6–8]. For instance, one study comparing an LB-endemic region of Switzerland to an LB-emerging region of Canada found that the proportion of individuals who reported performing tick checks after outdoor activities was approximately four-fold higher in those living in the endemic region compared to those living in the emerging region [8]. Another study of individuals living in endemic regions of Connecticut and Maryland in the United States found that higher self-rated knowledge of LB was significantly associated with performing tick checks [9]. Additionally, socioeconomic status, which is closely tied to geography [10], has been shown to impact both greenspace accessibility [11] and the prevalence of using tick-preventive behaviors [6, 9, 12]. However, the direction of the impact of socioeconomic status on LB risk remains uncertain, as previous studies have reported conflicting findings. Studies conducted in Denmark, Slovenia, the United Kingdom, and Canada have found associations between LB incidence and socioeconomic status, with higher income often linked to increased incidence [13–17]. Alternatively, a study in the United States found that lower socioeconomic status was associated with a higher incidence of disseminated LB (i.e., LB that has spread to other organ systems resulting in Lyme carditis, Lyme neuroborreliosis, etc.), possibly due to limited healthcare access leading to delayed diagnosis and treatment [18].

The possible factors influencing LB risk perception in Europe are not well described; an understanding of these could inform more effective targeting of prevention messages to populations most at risk of LB. Thus, the objective of this study was to consider potential factors shaping LB risk perception in the general adult population of twenty European countries, including urbanicity, socioeconomic status, gender, age, and past LB diagnosis history. The intent of this study was exploratory in nature and not aimed at establishing causality.

## Methods

### Study design and data

We conducted a cross-sectional survey using data obtained from an approximately fifteen-minute online survey taken by members of the general adult population (18–65 years old) in twenty European countries in 2022 [19]. Quality checks of completed surveys were conducted to identify and exclude responses from respondents with clear signs of speeding or providing random answers. The survey included questions on demographics (i.e., household income, gender, age, urbanicity, and household dog ownership), awareness of LB and ticks, and history of LB diagnosis and tick bites.

The survey was designed to provide representativeness estimates for the populations of the twenty countries included in the survey; participants were recruited with quotas placed on age, gender, and country subregion, and the final dataset was weighted on age, gender, and country subregion to approximate the population distribution for each country. All analyses were conducted on the weighted dataset. Additional details about the survey, including the sample size determination process, the sample size allocation by country, the representativeness of the sample, and the methods used to compute sampling weights can be found in Gould et al. [19]. An English language version of the complete survey instrument can be found in Additional File 1, Table S1.

The survey was determined exempt from institutional review board oversight by the study investigators in accordance with categories of exempt research under 45 CFR part 46.104, Exempt Research [20]. All research was compliant with the principles laid out in the Helsinki Declaration [21]. Participation in the survey questions was preceded by a notice of the voluntariness of the survey, the respondent’s right to withdraw at any point, an agreement about a provision of health data, and a notice of privacy policies. Respondents were asked to check boxes to confirm their understanding and agreement before proceeding to the survey questions.

### Measurement of LB risk perception (dependent variable)

LB risk perception was analyzed as an ordinal variable and was measured using a survey question that asked respondents, “How high would you estimate your risk is for contracting Lyme borreliosis?” Perceived risk was rated on a five-point scale, where one was defined as “very low risk” and five was defined as “very high risk.”

### Statistical analysis

Analyses only included respondents who knew their income information and who answered affirmatively to survey questions when asked whether they had heard of ticks and LB. The survey was designed such that those who had not heard of ticks or LB were not asked further questions about ticks and LB. However, all questions were mandatory unless skipped due to skip patterns in the survey logic. Socioeconomic status was determined from self-reported income, which was based on a survey question asking respondents to select from a range of income brackets. To account for variation in median incomes across countries, the data were standardized by categorizing income into three levels: low-income, middle-income, or high-income. Income levels were categorized by extracting the annual median income for each country from the Organization for Economic Co-operation and Development (OECD) Data Explorer’s income distribution database [22]. These medians were then used to create interquartile ranges from the lower- and upper-bounds of the survey’s income bracket response options (250 €–7001 € in the equivalent currency of a given country). Respondents at or below the 25th percentile were classified as low-income, respondents at or above the 75th percentile were classified as high-income, and respondents greater than the 25th percentile and less than the 75th percentile were classified as middle-income. High income was used as the reference group for comparisons between income levels. Urbanicity was categorized based on self-reported residence in a city (urban), in the suburbs of a city/outlying residential district of a city (suburban), or in the countryside (rural). (Existing sources have found self-reported urbanicity to have decent alignment with other objective measures of urbanicity in Europe [22]). Urban was used as the reference group for comparisons among urbanicity groups. Descriptive statistics, including weighted counts and weighted proportions, were calculated.

Adjusted odds ratios (AOR) were obtained from a regression analysis using the five-point scale LB risk perception variable described above. Because the outcome variable consisted of greater than two levels with an inherent order (i.e., from lowest to highest risk perception), an ordinal logistic regression model was selected to evaluate relationships between predictor variables and LB risk perception. To meet the assumptions of ordinal logistic regression, we assumed proportional odds (i.e., the notion that the odds of being in a higher versus lower category of risk perception is consistent across all thresholds of the ordinal outcome) and no multi-collinearity. To evaluate the assumption of proportional odds, we fit separate survey-weighted logistic regression models for each level of the LB risk perception outcome variable. This allowed us to assess the odds of having an LB risk perception score of one versus two to five, one or two versus three to five, and so on. We tested the assumption of no multi-collinearity by performing a variance inflation factor (VIF) test, using a VIF value of ten as a threshold for collinearity, as other sources have suggested this is a reasonable cutoff [23].

The regression model was adjusted for urbanicity, income level, age, gender, tick bite history, dog ownership, and LB diagnosis history (in oneself and in someone respondents knew). We also included interaction terms between urbanicity and income level and urbanicity and LB diagnosis history.

Variable selection was determined from a review of published literature suggesting that urbanicity, age, gender, tick bite history, dog ownership, and LB diagnosis history may influence LB risk perception [6–8, 23, 24]. We also included income status in the model, as it may be a proxy variable for several other risk factors, e.g., access to greenspace, living in homes with yards, ability to travel and engage in outdoor leisure activities. Interaction terms were incorporated to explore how urbanicity might modify the relationship between income level and LB risk perception and the relationship between past LB infection and LB risk perception. These interaction terms were selected because urbanicity may influence environmental exposure (e.g., through greenspace availability), access to healthcare, and awareness of LB risk, potentially impacting how income level and past infection shape risk perception.

All analyses were performed using R Statistical Software (R version 4.4.1; R Core Team 2024) and the R survey package (version 4.2, Lumley 2023) to account for the survey design and weighting. Weighted survey data were used throughout the analysis, including in the regression model. Significance was considered a *p*-value of < 0.05.

## Results

**Fig. 1:**
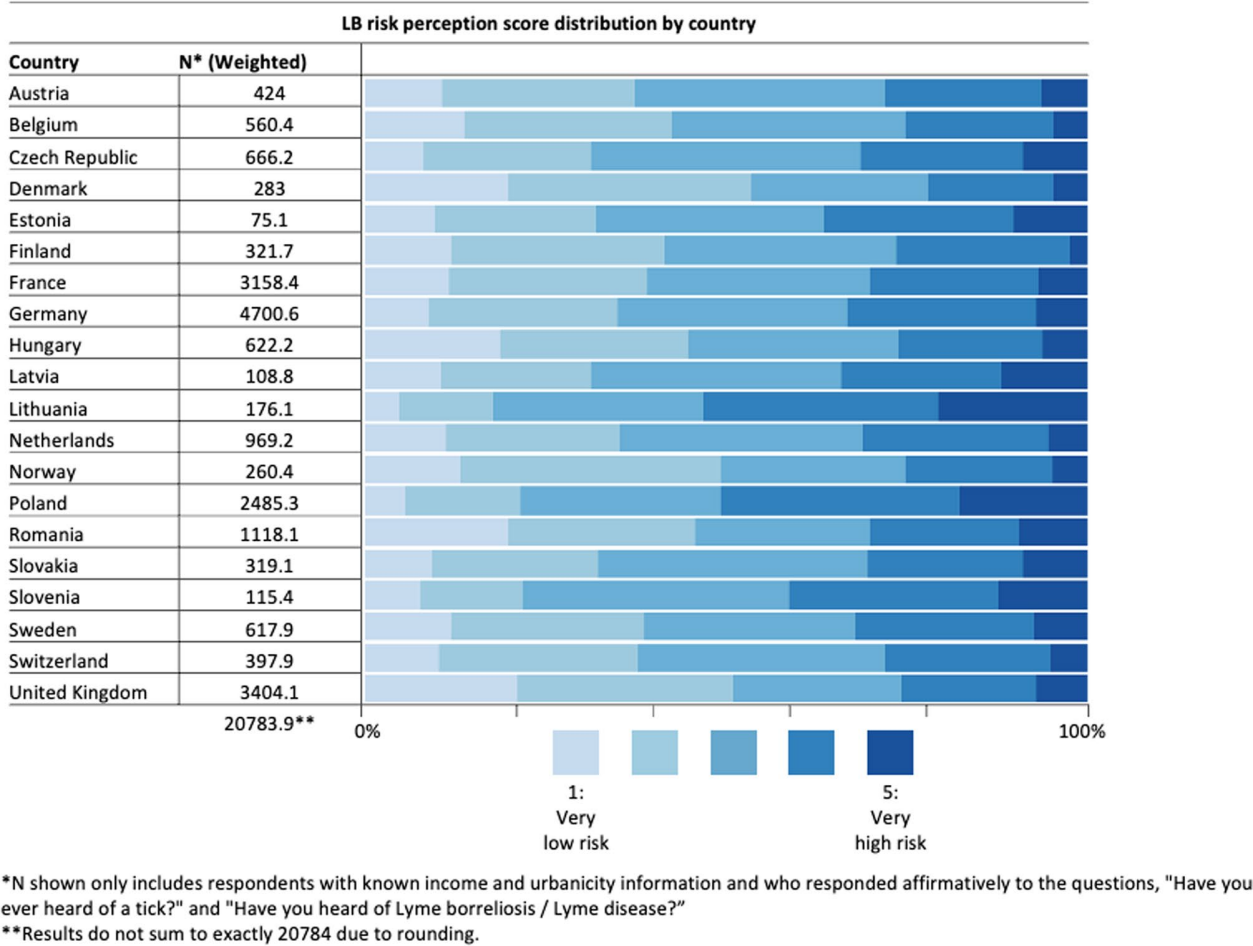
Lyme borreliosis (LB) weighted sample sizes and survey respondent risk perception scores by country (the width of the bar represents the weighted percentage of respondents in a given risk perception category); results are weighted by country subregion, gender, and age

A total of 28,034 adults (aged 18–65) from 20 countries completed surveys. Of these 28,034 respondents, 85% had heard of ticks and LB and thus did not have any missing data due to skip patterns. The final analytic sample consisted of 20,784 (weighted count) respondents who met the full inclusion/exclusion criteria (i.e., reported having heard of ticks and LB and did not respond with “unknown” to the income and urbanicity questions). A complete list of the twenty countries included and of the number of respondents from each country making up the final analytic sample can be found in Fig. 1.

Respondents were most likely to be female (51.4%, 95% confidence interval [CI]: 50.5–52.3%), aged 50–65 (37.9%, 95% CI: 37.0–38.8%), reside in urban areas (46.8%, 95% CI: 45.9–47.7%), and be of middle-income (55.0%, 95% CI: 54.1–55.9%). About half of all respondents reported ever having a tick bite (51.4%, 95% CI: 50.5–52.3%), and 8.6% (95% CI: 8.1–9.1%) reported ever personally experiencing a diagnosis of LB (with 38.9%, 95% CI: 38.0–39.8%, reporting knowing someone who ever had an LB diagnosis) (Table 1).

**Table 1:** Lyme borreliosis (LB) survey respondent baseline characteristics from Twenty countries in europe, 2022; proportions and counts have been weighted by country subregion, gender, and age*

Characteristic	Overall *N* = 20,784 (100%)	95% Confidence Interval
Gender		
Female	10,686 (51.4%)	(50.5–52.3%)
Male	10,057 (48.4%)	(47.5–49.3%)
Age		
18–29	3,709 (17.8%)	(17.1–18.5%)
30–39	4,521 (21.7%)	(21.0–22.5%)
40–49	4,673 (22.5%)	(21.7–23.3%)
50–65	7,880 (37.9%)	(37.0–38.8%)
Urbanicity		
Urban	9,738 (46.8%)	(45.9–47.7%)
Suburban	5,691 (27.4%)	(26.6–28.2%)
Rural	5,355 (25.8%)	(25.0–26.6%)
Income status		
High	4,997 (24.1%)	(23.3–24.9%)
Middle	11,431 (55.0%)	(54.1–55.9%)
Low	4,355 (20.9%)	(20.2–21.7%)
Household dog ownership	8,513 (41.0%)	(40.1–41.9%)
Ever had a tick bite	10,690 (51.4%)	(50.5–52.3%)
Ever personally had an LB diagnosis	1,788 (8.6%)	(8.1–9.1%)
Knows someone who ever had an LB diagnosis	8,084 (38.9%)	(38.0–39.8%)

*Weighted counts have been rounded to the nearest whole number and therefore may not sum exactly to the N shown

**Fig. 2:**
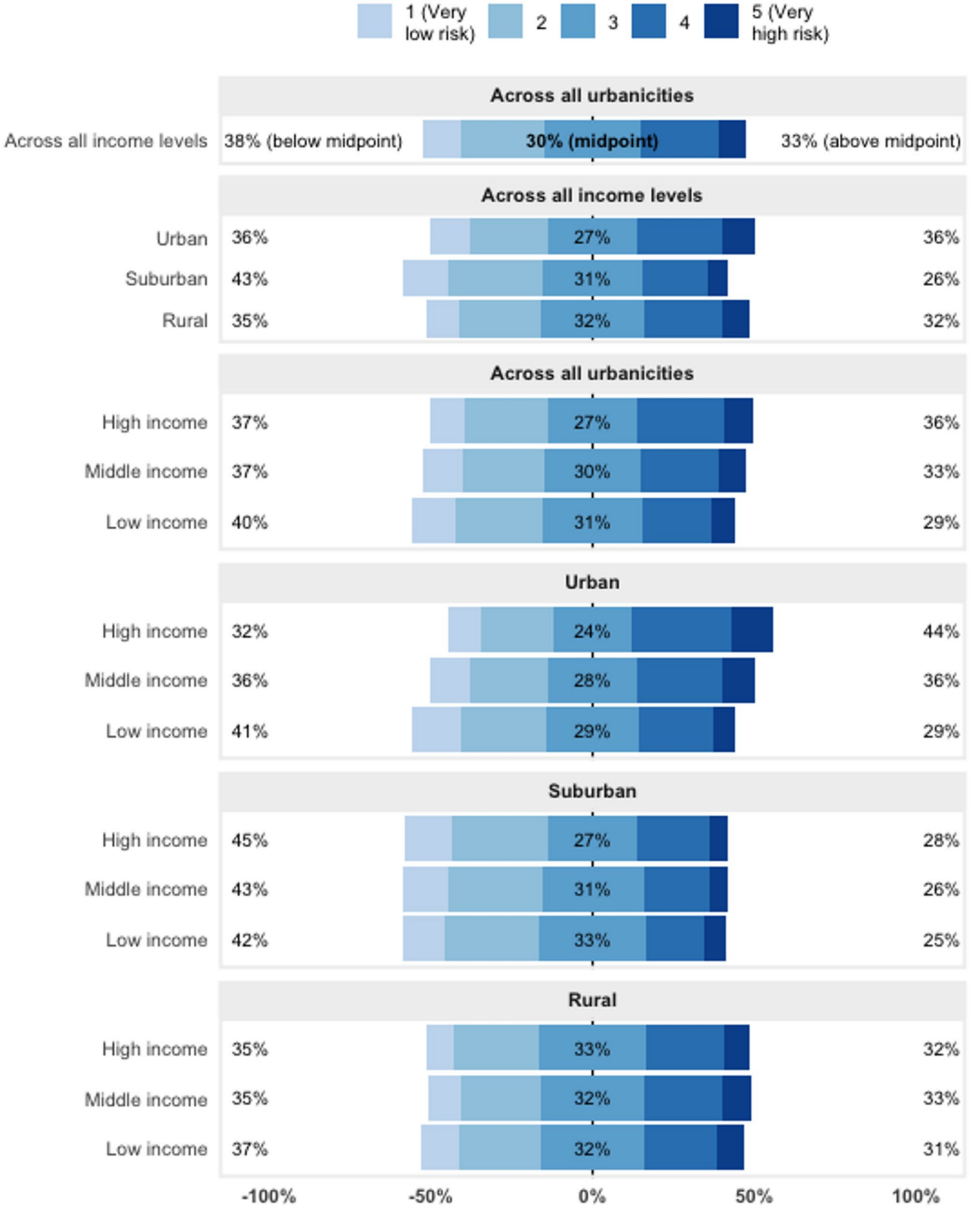
Likert scale plot of Lyme borreliosis risk perception scores from respondents in a multi-country survey from twenty countries in Europe, 2022, results are shown with and without stratifications by urbanicity and income level; proportions have been weighted by country subregion, gender, and age

Among income groups, low-income respondents had the lowest LB risk perception scores (40.4% rated with a score of one or two, 95% CI: 38.1–42.7%, as compared to 37.5%, 95% CI: 36.0-38.9% of middle-income and 37.5%, 95% CI: 34.6–39.0% of high-income respondents), whereas high-income respondents had the highest LB risk perception scores (36.8%, 95% CI: 33.8–38.2% rated with a four or five as compared to 32.5%, 95% CI: 31.0–34.0%, of middle-income and 28.7%, 95% CI: 26.2–31.2%, of low-income respondents) (Fig. 2). When considering all possible combinations of urbanicity and income level, respondents who were high-income and suburban had the lowest risk perception scores (45% rated with a score of one or two, 95% CI: 39.8–49.4%), whereas respondents who were high-income and urban had the highest LB risk perception scores (44% with a score of four or five, 95% CI: 39.7–47.6%) (Fig. 2). At the country level, Lithuania had the highest LB risk perception scores (53.2% with a score of four or five, 95% CI: 50.2–56.2%), whereas Denmark had the lowest LB risk perception scores (53.4% with a score of one or two, 95% CI: 49.7–57.2%) (Fig. 1).

### Regression analysis

Results from the VIF test to assess collinearity can be found in Additional File 1, Table S2. No VIF values fell above 5.25, providing assurance that the model was within the predefined threshold of 10. The proportional odds assumption held reasonably well; coefficient estimates across cumulative logit models remained stable, with no deviations exceeding 1 on the log-odds scale for any predictor. Personally having a past LB diagnosis (AOR: 3.96, 95% CI: 3.38–4.64) was most closely tied to heightened LB risk perception. Suburban residence (AOR: 0.72, 95% CI: 0.61–0.86), younger age (aged 18–29) (AOR: 0.88, 95% CI: 0.80–0.97), and male gender (AOR: 0.79, 95% CI: 0.74–0.84) were also associated with lower LB risk perception. Conversely, the 30–39-year (AOR: 1.34, 95% CI: 1.22–1.46) and 40–49-year (AOR: 1.17, 95% CI: 1.07–1.28) age groups were positively associated with higher LB risk perception (Table 2).

**Table 2:** Ordinal logistic regression model of the outcome, Lyme borreliosis (LB) risk perception, adjusted for income level, urbanicity, age, gender, household dog ownership, tick bite history, and LB diagnosis history with interaction terms between urbanicity and income level and urbanicity and past LB diagnosis; *P-value < 0.05; **P-value < 0.001

Predictor	Adjusted Odds Ratios (95% CI)	*P*-value
Income level		
High (reference)		
Middle income	1.03 (0.91–1.16)	0.663
Low income	0.82 (0.70–0.95)	0.009*
Urbanicity		
Urban (reference)		
Suburban	0.72 (0.61–0.86)	< 0.001**
Rural	0.96 (0.81–1.14)	0.636
Age		
50–65 (reference)		
18–29	0.88 (0.80–0.97)	0.011*
30–39	1.34 (1.22–1.46)	< 0.001**
40–49	1.17 (1.07–1.28)	< 0.001**
Gender		
Female (reference)		
Male	0.79 (0.74–0.84)	< 0.001**
Owns a household dog	1.42 (1.33–1.53)	< 0.001**
Ever had a tick bite	1.71 (1.60–1.84)	< 0.001**
LB diagnosis history		
Ever personally had an LB diagnosis	3.96 (3.38–4.64)	< 0.001**
Knows someone who ever had an LB diagnosis	2.11 (1.97–2.27)	< 0.001**
INTERACTION TERMS		
Urbanicity × Income level		
Suburban urbanicity × Middle income	0.81 (0.70–0.93)	0.003*
Rural urbanicity × Middle income	0.92 (0.81–1.07)	0.294
Suburban urbanicity × Low income	0.81 (0.68–0.97)	0.023*
Rural urbanicity × Low income	0.91 (0.76–1.09)	0.311
Urbanicity × Past LB diagnosis		
Suburban urbanicity × Past LB diagnosis	2.86 (2.09–3.90)	< 0.001**
Rural urbanicity × Past LB diagnosis	4.92 (3.67–6.60)	< 0.001**

Significant interaction effects were observed for suburban respondents of middle income (AOR: 0.81, 95% CI: 0.70–0.93) and suburban respondents of low income (AOR: 0.81, 95% CI: 0.68–0.97), suggesting that the association between suburban urbanicity and LB risk perception may vary by income level, with suburban middle- and low-income groups demonstrating a lower odds of LB risk perception compared to other urbanicity and income groups. There were also interactions between urbanicity and a previous diagnosis of LB (Suburban urbanicity × Past LB diagnosis AOR: 2.86, 95% CI: 2.09–3.90; Rural urbanicity × Past LB diagnosis AOR: 4.92, 95% CI: 3.67–6.60), suggesting that the relationship between urbanicity and LB risk perception varies by past LB diagnosis status. Specifically, individuals living in suburban and rural areas reported higher LB risk perception odds if they had a prior diagnosis of LB, with this effect being especially pronounced among rural respondents (Table 2).

Crude odds ratios can be found in the supplement (Additional File 1, Table S3).

## Discussion

This multi-country survey study provides valuable insight into the factors that shape LB risk perception in the European general adult population. Having a past diagnosis of LB was the strongest predictor of LB risk perception. Other factors, including suburban residence, being between 30 and 49 years of age, owning a dog, and knowing someone with a past LB diagnosis were also predictive of risk perception. High-income respondents were more likely to report a past tick bite (55.4%, 95% CI: 53.5–57.3%, compared to 51.1%, 95% CI: 49.9–52.3%, and 48.0%, 95% CI: 45.7–49.7%, of middle- and low-income respondents, respectively; results not shown). High-income respondents were also more likely to report a past LB diagnosis than middle- and low-income respondents (13.5%, 95% CI: 12.1–14.8% compared to 6.4%, 95% CI: 5.8–6.9%, and 8.9%, 95% CI: 7.8–10.0%, of middle- and low-income respondents, respectively; results not shown).

The highest level of LB risk perception was observed in those with a past LB diagnosis. A past tick bite was also associated with higher risk perception, though to a lesser extent. Assuming that engaging in preventive behaviors is an objective manifestation of risk perception, these findings are supported by other studies, which have found that prior diagnoses of tick-borne disease and past tick bites are associated with preventive behaviors. For instance, one study from the Netherlands that sought to assess predictors of using LB preventive measures found greater odds of always or often checking skin for the presence of ticks to prevent Lyme disease in those with a past tick bite [12]. Similarly, a Canadian study found previous infection with LB and previous tick bites to be strongly associated with the adoption of protective behaviors [6]. In the United States, another study found that individuals with a past tick-borne disease diagnosis were more likely to conduct tick checks and shower or bathe after spending time outdoors [9]. Collectively, these studies underscore how personal experience with ticks and prevention behaviors influence one another (e.g., presumedly more tick checks lead to more realized tick bites and more realized tick bites lead to more future tick checks) while also shaping risk perception. However, it should be noted that these are likely not the only important factors, as findings from this study and others suggest that variables such as gender, age, and dog ownership, among others, may also play a role in shaping LB risk perception [6–8, 23, 24].

Prior studies from Scandinavia, the United States, and Canada have similarly reported higher risk perception for LB among females and dog owners and lower risk perception among younger adults (especially 18–29-year-olds) [8, 23, 24]. The Scandinavian study found that previous diagnosis of LB was associated with lower perceptions of LB severity. This finding likely reflects the fact that “perceived severity” captures a different attribute than the “perceived risk of contracting LB.” A prior analysis of these data found that most respondents rated Lyme disease as severe or very severe (79.3%, 95% CI: 78.6–80.0%). In contrast, only 32% (95% CI: 31.2–32.8%) said they perceived themselves to be at high or very high risk of contracting LB. Both perceived severity and perceived risk of contracting risk of LB were higher among persons with a past LB diagnosis, although less so for severity [19]. Future studies exploring the role of perceived severity in the uptake of prevention behaviors could be a meaningful contribution to the growing body of research on LB risk perception.

Higher reported rates of tick bites and LB infection in high-income individuals are consistent with several past studies, including a case-control study from Canada, a healthcare database study from Denmark, an ecological study from the United States, and an analysis of reported cases in New York City, which found human LB infection to be associated with residence in counties of higher socioeconomic status [13–15, 25]. These results might reflect residence in homes with larger yards and green spaces for recreational use, increasing the probability of tick encounters. Independent of residence, persons of higher income might also be more likely to engage in travel and outdoor leisure activities that put them in contact with infected ticks; this is supported by an analysis of the analytic dataset used in the study, which demonstrated that high-income individuals were more likely than middle- and low-income respondents to report engaging in outdoor activities such as wilderness backpacking (See Additional File 1, Table S4). In the same analysis, urban respondents similarly reported being more likely to engage in such outdoor activities compared to suburban and rural respondents. This may reflect the fact that among high-income respondents, urban residence was the most common urbanicity setting reported (Fig. 2). Additional statistics describing time respondents spent outdoors can be found in Gould et al. [19].

Significant interaction effects in our study suggest that the association between suburban urbanicity and LB risk perception may vary by income level, with suburban middle- and low-income groups having a lower odds of LB risk perception compared to other urbanicity and income groups. There is likely a nuanced relationship between urbanicity and socioeconomic status on LB risk perception, perhaps because of differences in outdoor exposure, LB awareness, or other factors that were not accounted for in this analysis.

Interactions between urbanicity and previous LB diagnosis status also suggested that individuals living in suburban and rural areas may have higher LB risk perception if they had a prior diagnosis of LB, as compared to their urban counterparts. Perhaps urbanicity influences community-level awareness, with greater LB awareness afforded to residents of non-urban areas—potentially leading to fewer undiagnosed cases and higher LB risk perception. We cannot determine to what extent this may or may not be the case from our study alone, but it may be an interesting topic of future research.

While the interaction effects present in this study should be interpreted at the pan-European level, they underscore a need for future studies making regional comparisons. The differing influence of income and prior LB diagnosis across urbanicity categories suggests that regional variation in healthcare access, personal income, or environmental exposure might also shape risk perception.

Our study has several limitations, including the risk of selection bias and recall bias from self-reported data obtained from an existing survey panel. Quotas and weighting based on gender, age, and country subregion aimed to mitigate issues due to a potential lack of representativeness, but we cannot rule out the possibility of unmeasured confounding. Additionally, the survey excluded adults over 65 years of age, so if LB risk perception differs in this age group (which is at a higher risk of developing LB infection than younger adults in the general population [26, 27]), compared to the rest of the study sample, this would not have been captured by our study. Furthermore, the LB risk perception outcome was based on a survey question that was only asked of respondents who knew what ticks and LB were. While high proportions of survey respondents reported awareness of ticks and LB (95%, 95% CI: 94.6–95.4% and 85%, 95% CI: 84.5–86.0%, respectively), the small percentage of respondents who were unaware and not asked about their perceptions of risk would presumably have lower LB risk perception scores, given their lack of awareness. Future surveys may consider including more granular questions about knowledge of ticks and LB so that a more comprehensive assessment of how awareness influences risk perception can be conducted. Finally, our study sought to examine predictors of LB risk perception broadly across Europe. Future studies could build upon our findings by exploring country-specific differences in LB risk perception. Additional country-level results using the same survey data may also be found in Gould et al. [19].

## Conclusions

This European multi-country study of the general adult population provides valuable data on the factors that shape LB risk perception. Such understanding is critical because LB risk perception could influence the adoption and continued use of preventive behaviors to protect against tick bites and LB. Insight into these motivating factors can inform targeted prevention messages and policies.

## Supplementary Information

Supplementary Material 1.

## Data Availability

The data that support the findings of this study are available from the corresponding author, Emily Colby, upon reasonable request.
