# Supplementary material for: Factors influencing Lyme borreliosis risk perception in Europe: a cross-sectional multi-country survey study
Source: BMC Public Health. 2025 Aug 1;25:2603. doi: 10.1186/s12889-025-23722-z (PMC12315446; doi:10.1186/s12889-025-23722-z)
Supplement: Supplementary file 1 — Supplementary Material 1. [file 12889_2025_23722_MOESM1_ESM.docx]

**Supplementary Material**

**Journal:** *BMC Public Health*

**Manuscript title:** Factors Influencing Lyme Borreliosis Risk Perception in Europe: A Cross-Sectional Multi-Country Survey Study

**Authors:** Emily Colby, L. Hannah Gould, Ye Tan, Andreas Pilz, Gordon Brestrich, Jennifer C. Moisi, and James H. Stark

*(Please see main text file for full affiliation list)*

**Contents (the table legends listed below are also listed at the top of each respective table in the subsequent pages)**

**Table S1.** Full questionnaire used in multi-country Lyme borreliosis survey in 20 European countries

**Table S2.** Assessment of variation inflation factors (VIF) of independent predictor variables

**Table S3.** Crude odds ratio estimates evaluating predictor variables for Lyme borreliosis (LB) risk perception

**Table S4.** Outdoor activity engagement by income status and urbanicity; counts and proportions have been weighted by country region, gender, and age

**Table S1.** Full questionnaire used in multi-country Lyme borreliosis survey in 20 European countries

**Master Questionnaire**

**Lyme Risk Survey – Europe**

Austria, Belgium, Czech Republic, Denmark, Estonia, Finland, France, Germany, Hungary, Latvia, Lithuania, Netherlands, Norway, Poland, Romania, Slovakia, Slovenia, Sweden, Switzerland, UK

[Introduction to the survey, incl. privacy policy.]

**Region – List provided by country**

**Base: All**

E01 [S]

In which region do you live?

**Age**

**Base: All**

A01 [Q]

Please indicate your age.

______ years

*SCRIPTER: Screen out if <18 y.o. or >65 y.o.*

**Gender**

**Base: All**

A02 [S]

Please indicate your gender.

1. Male
2. Female
3. Other
4. Prefer not to answer

**Children in household**

**Base: All**

A03 [Q]

How many children under the age of 18 years live in your household (at least part time)?

Number of children in household: _____

*SCRIPTER: Provide options 0-15 in dropdown menu*

**Tick awareness**

**Base: All**

B01 [S]

Have you ever heard of “a tick” – a small blood sucking bug living in green areas?

1. Yes
2. No
3. Don’t know

**Tick in country**

**Base: All**

B02 [S]

Do you think ticks live in your country?

1. Yes
2. No
3. Don’t know

**Tick in local area**

**Base: B02≠2**

B03 [S ]

Do you think ticks are present in the county or district where you live?

1. Yes
2. No
3. Don’t know

**Risk of tick bite, personal**

**Base:** **All tick aware (B01=1 OR B02=1 OR B03=1)**

B04 [Rating slider]

How high would you estimate your risk is for being bitten by a tick?

Please use the scale from 1 to 5, where 1 is “Very low risk” and 5 is “Very high risk”.


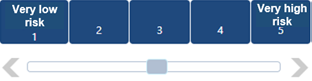


**Child´s risk of tick bite**

**Base: All with at least 1 child (A03>0) and tick aware (B01=1 OR B02=1 OR B03=1)**

B05 [Rating slider]

How high would you estimate the risk is for your child(ren) being bitten by a tick?

Please use the scale from 1 to 5, where 1 is “Very low risk” and 5 is “Very high risk”.


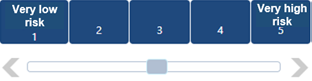


**Tick bite prevention**

**Base: All tick aware (B01=1 OR B02=1 OR B03=1)**

B06 [Rating grid slider]

How often do you use the below preventative measures to help protect you against tick bites.

Please use the scale from “Always” to “Never”.

1. Wear protective clothing / long socks / tuck trouser legs into socks
2. Use insect repellent such as DEET
3. Avoid tick-infested areas
4. Check for ticks after spending time outside
5. Other

*SCRIPTER: Randomize 1-4, anchor 97*

*SCRIPTER: Rating slider: Always/Often/Sometimes/Seldom/Never/I don´t know*

**Tick bite, personal, ever**

**Base: All tick aware (B01=1 OR B02=1 OR B03=1)**

B07 [S]

Have you ever been bitten by a tick?

1. Yes
2. No
3. I don’t know

**Tick bite, personal, last year**

**Base: All tick aware (B01=1 OR B02=1 OR B03=1) and bitten by tick (B07=1)**

B08 [Q]

During the last year, how many times have you been bitten by a tick, if at all?

____times during the last year

**Tick bite, child, ever**

**Base: All tick aware (B01=1 OR B02=1 OR B03=1) and with at least 1 child (A03>0)**

B09a [S]

Among the children under the age of 18 years living in your household (at least part time), please now think of the oldest. Have they ever been bitten by a tick?

1. Yes
2. No
3. I don’t know

**Tick bite, child, last year**

**Base: All tick aware (B01=1 OR B02=1 OR B03=1) and w 1+ child (A03>0) , ever bitten (B09a=1)**

B09 [Q]

Please again think of the oldest child under the age of 18 years living in your household. During the last year, how many times have they been bitten by a tick, if at all?

____times during the last year

**Tick Worry**

**Base: All tick aware (B01=1 OR B02=1 OR B03=1)**

B10 [Rating slider, per statement]

Please rate your agreement with the following statements:

1. I worry about ticks, independent of whether they carry disease
2. I only worry about ticks because the diseases they transmit can be severe

Please use the scale from 1 to 5, where 1 is “Disagree strongly” and 5 is “Agree strongly”.


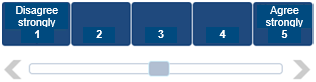


*SCRIPTER: Randomize statements*

**Lyme awareness**

**Base: All tick aware (B01=1 OR B02=1 OR B03=1)**

C01 [S per row]

Which of the following diseases have you ever heard of?

For each, please select ´Yes´ or ´No´ to reflect your opinion.

1. Anaplasmosis
2. Lyme borreliosis / Lyme disease
3. Tick-borne encephalitis (TBE)
4. None of the above [S]
5. Don’t know [S]

*SCRIPTER: Randomize 1-3*

*SCRIPTER: In columns: Yes/No/I don’t know*

**Lyme disease in local area**

**Base: All tick aware (B01=1 OR B02=1 OR B03=1) and aware of Lyme disease (C01=2)**

C02 [S]

Do you think Lyme borreliosis exists in the county or district where you live and/or work?

1. Yes
2. No
3. I don’t know

**Own risk of Lyme disease**

**Base: All tick aware (B01=1 OR B02=1 OR B03=1) and aware of Lyme disease (C01=2)**

C03 [Rating slider]

How high would you estimate your risk is for contracting Lyme borreliosis?

Please use the scale from 1 to 5, where 1 is “Very low risk” and 5 is “Very high risk”.


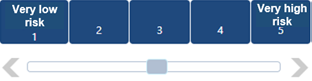


**Concern about contracting Lyme disease**

**Base: All tick aware (B01=1 OR B02=1 OR B03=1) and aware of Lyme disease (C01=2)**

C04 [Rating slider]

How concerned are you about contracting Lyme borreliosis?

Please use the scale from 1 to 5, where 1 is “Not at all concerned” and 5 is “Extremely concerned”.


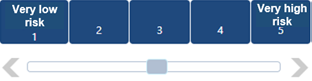


**Child´s risk of Lyme disease**

**Base: All tick aware (B01=1 OR B02=1 OR B03=1) w 1+ child (A03>0), and aware of LD (C01=2)**

C05 [Rating slider]

How high would you estimate the risk is for your child(ren) contracting Lyme borreliosis?

Please use the scale from 1 to 5, where 1 is “Very low risk” and 5 is “Very high risk”.


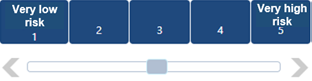


**Concern about child contracting Lyme disease**

**Base: All tick aware (B01=1 OR B02=1 OR B03=1) w 1+ child (A03>0), and aware of LD (C01=2)**

C05b [Rating slider]

How concerned are you about your child(ren) contracting Lyme borreliosis?

Please use the scale from 1 to 5, where 1 is “Not at all concerned” and 5 is “Extremely concerned”.


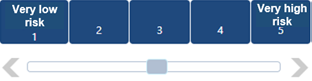


**Lyme disease diagnosis**

**Base: All tick aware (B01=1 OR B02=1 OR B03=1) and aware of Lyme disease (C01=2)**

C06 [S]

Have you ever been told by a doctor that you had Lyme borreliosis?

1. Yes
2. No
3. I don’t know

**Severity of own Lyme disease**

**Base: Had Lyme disease (C06=1)**

C06b [S]

You mentioned you were diagnosed with Lyme borreliosis. Was it a severe disease?

1. Yes
2. No
3. I don’t know

**Lyme disease peers**

**Base: All tick aware (B01=1 OR B02=1 OR B03=1) and aware of Lyme disease (C01=2)**

C07 [S]

Do you personally know anyone who had Lyme borreliosis?

1. Yes
2. No
3. I don’t know

**Severity of peer´s Lyme disease**

**Base: Peer had Lyme disease (C07=1)**

C08 [S]

You mentioned to know someone who had Lyme borreliosis. Was it a severe disease?

1. Yes
2. No
3. I don’t know

**Lyme disease severity - % of cases**

**Base: All tick aware (B01=1 OR B02=1 OR B03=1) and aware of Lyme disease (C01=2)**

C09 [Q]

What proportion of Lyme borreliosis cases would you expect to have severe symptoms?

_ _ _ % of Lyme borreliosis cases have severe symptoms

1. I don’t know

**Lyme disease severity - scale**

**Base: All tick aware (B01=1 OR B02=1 OR B03=1) and aware of Lyme disease (C01=2)**

C10 [Rating slider]

How severe do you think Lyme borreliosis is?

Please use the scale from 1 to 5, where 1 is “Not severe at all” and 5 is “Extremely severe”.


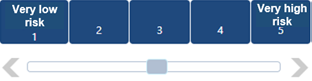


**Lyme disease manifestation**

**Base: All tick aware (B01=1 OR B02=1 OR B03=1) and aware of Lyme disease (C01=2)**

C12 [Multi Grid – Drag and Drop]

What symptom(s) can a person infected by Lyme borreliosis have?

Please place each symptom into the relevant group of “Can be symptom of Lyme borreliosis”, “Is not a Lyme borreliosis symptom”, or “I don´t know”.

1. Rash (erythema migrans)
2. Joint pain (arthritis)
3. Neurological problems
4. Heart problems
5. Coughing
6. Blocked nose
7. Diarrhea
8. Severe fatigue


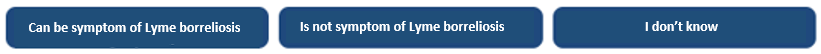
*SCRIPTER: Randomize symptoms*

**Expected duration of Lyme**

**Base: All tick aware (B01=1 OR B02=1 OR B03=1) and aware of Lyme disease (C01=2)**

C11 [S]

How long would you expect the symptoms of Lyme borreliosis to last?

1. Some days
2. Some weeks
3. Some months
4. Some years
5. I don´t know

**Heard of Lyme in media**

**Base: All tick aware (B01=1 OR B02=1 OR B03=1) and aware of Lyme disease (C01=2)**

C14 [Rating grid slider]

How often have you heard of Lyme borreliosis in the media?

Please use the scale from “Always” to “Never”.

*SCRIPTER: Rating slider: Always/Often/Sometimes/Seldom/Never/I don´t know*

**Talk about Lyme with peers**

**Base: All tick aware (B01=1 OR B02=1 OR B03=1) and aware of Lyme disease (C01=2)**

C15 [Rating grid slider]

How often have you discussed Lyme borreliosis with friends, family or acquaintances?

Please use the scale from “Always” to “Never”.

*SCRIPTER: Rating slider: Always/Often/Sometimes/Seldom/Never/I don´t know*

**Lyme risk statement battery**

**Base: All tick aware (B01=1 OR B02=1 OR B03=1) and aware of Lyme disease (C01=2)**

C16 [S, per statement, show one statement at a time]

Please rate your agreement with each of the below statements about risk of contracting Lyme borreliosis.

1. Living with children means a higher risk of contracting Lyme borreliosis
2. Having a dog means a higher risk of contracting Lyme borreliosis
3. Living in an area with Lyme borreliosis cases means a higher risk of contracting it
4. Spending time in green outdoor areas means a higher risk of contracting Lyme borreliosis

Please use the scale from 1 to 5, where 1 is “Disagree strongly” and 5 is “Agree strongly”.


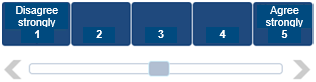


*SCRIPTER: Randomize statements*

**Perceived Severity – Statements**

**Base: All tick aware (B01=1 OR B02=1 OR B03=1) and aware of Lyme disease (C01=2)**

C17 [Rating slider, show one statement at a time]

Please rate your agreement with the following statements:

1. Contracting Lyme borreliosis would not have major consequences on my life
2. It would be very serious if I contracted Lyme borreliosis

Please use the scale from 1 to 5, where 1 is “Disagree strongly” and 5 is “Agree strongly”.


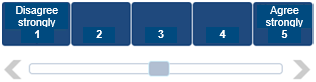


*SCRIPTER: Randomize statements*

**Likelihood of vaccination**

**Base: All tick aware (B01=1 OR B02=1 OR B03=1) and aware of Lyme disease (C01=2)**

C18 [Rating slider, show all statements on same screen]

If a vaccine was available against Lyme borreliosis and it was safe and effective, how likely would you be to get it if:

1. It was free of cost for you
2. Your health care provider recommended it
3. It was free of cost for you AND your health care provider recommended it

Please use the scale from 1 to 5, where 1 is “Very unlikely” and 5 is “Very likely”.


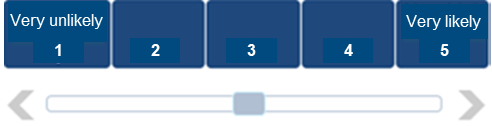


*SCRIPTER: Randomize 1-2, anchor 3*

*SCRIPTER: Do not show in FI, NL*

**Cues to action**

**Base: All tick aware (B01=1 OR B02=1 OR B03=1) and aware of Lyme disease (C01=2)**

C19 [Rating slider, show one statement at a time]

Please rate your agreement with the following statement: The chances of me getting vaccinated against Lyme borreliosis will increase if..:

1. People I follow on social media express support for the benefit of the vaccine
2. Friends and family express support for the benefit of the vaccine
3. Official guidelines from the health authorities are published
4. My health care provider recommends it to me
5. My workplace offers me the vaccination

Please use the scale from 1 to 5, where 1 is “Disagree strongly” and 5 is “Agree strongly”.


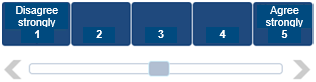


*SCRIPTER: Randomize statements*

*SCRIPTER: Do not show in FI, NL*

*SCRIPTER: Do not show Code 5 in HU*

**Barriers**

**Base: All tick aware (B01=1 OR B02=1 OR B03=1) and aware of Lyme disease (C01=2)**

C20 [Rating slider, show all statements on one sreen]

How much would the following factors prevent you from getting a hypothetical vaccine against Lyme borreliosis?

1. If you have an out-of-pocket cost
2. Concerns about possible side effects of the vaccine
3. Concerns about whether the vaccine is safe

Please use the scale from 1 to 5, where 1 is “Disagree strongly” and 5 is “Agree strongly”.


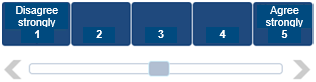


*SCRIPTER: Randomize statements*

*SCRIPTER: Do not show in FI, NL*

**Benefits**

**Base: All tick aware (B01=1 OR B02=1 OR B03=1) and aware of Lyme disease (C01=2)**

C21 [Rating slider, show all statements on same screen]

Please rate your agreement with the following statements:

If a vaccine for Lyme borreliosis existed and I were to get it..

1. I will reduce my risk of getting infected with Lyme borreliosis
2. I will decrease my chances of getting sick with Lyme borreliosis
3. I will reduce my chances of getting a severe form of Lyme borreliosis

Please use the scale from 1 to 5, where 1 is “Disagree strongly” and 5 is “Agree strongly”.


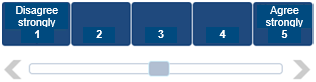


*SCRIPTER: Randomize statements*

*SCRIPTER: Do not show in FI, NL*

**Self-efficacy**

**Base: All tick aware (B01=1 OR B02=1 OR B03=1) and aware of Lyme disease (C01=2)**

C22 [Rating slider, show all statements on same screen]

Please assume a vaccine against Lyme borreliosis was safe, effective and available to you.

Please rate your agreement with the following statements:

1. I am capable of getting the vaccine even if it is expensive
2. I am capable of getting the vaccine even if it means finding time to go to the doctor multiple times to receive all required doses to be protected
3. I would **not** be capable of getting the vaccine if the injection hurts a little
4. I would **not** get the vaccine, because the risk of the vaccine is higher than the risk of the disease

Please use the scale from 1 to 5, where 1 is “Disagree strongly” and 5 is “Agree strongly”.


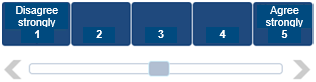


*SCRIPTER: Anchor 1-2 and 3-4, rotate order between each and among the two set*

*SCRIPTER: Do not show in FI, NL*

**Inaction Regret**

**Base: All tick aware (B01=1 OR B02=1 OR B03=1) and aware of Lyme disease (C01=2)**

C23 [Rating slider]

Please rate your agreement with the following statements:

1. If I did not get the Lyme borreliosis vaccine when offered, but three months later contracted the disease, I would strongly regret declining the opportunity

Please use the scale from 1 to 5, where 1 is “Disagree strongly” and 5 is “Agree strongly”.


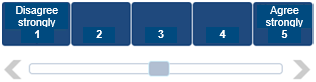


*SCRIPTER: Do not show in FI, NL*

**Tick Disgust**

**Base: All tick aware (B01=1 OR B02=1 OR B03=1)**

C24 [Rating slider, show all statements on same screen]

Please rate your agreement with the following statements:

I would feel disgust while walking outdoors, if...

1. I saw a real ladybug on my friend's clothing
2. I saw a real spider on my friend's clothing
3. I saw a tick on my friend's clothing

Please use the scale from 1 to 5, where 1 is “Disagree strongly” and 5 is “Agree strongly”.


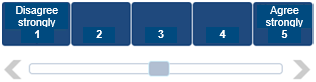


*SCRIPTER: Randomize statements*

**Perceived susceptibility**

**Base: All tick aware (B01=1 OR B02=1 OR B03=1) and aware of Lyme disease (C01=2)**

C25 [Rating slider, show all statements on same screen]

Please rate your agreement with the following statements:

If a vaccine for Lyme borreliosis existed, and I did **not** get vaccinated…

1. I am likely to get Lyme borreliosis
2. I would feel vulnerable to getting Lyme borreliosis sometime in the future

Please use the scale from 1 to 5, where 1 is “Disagree strongly” and 5 is “Agree strongly”.


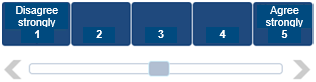


*SCRIPTER: Randomize statements*

*SCRIPTER: Do not show in FI and NO, NL*

**Confidence/Control**

**Base: All tick aware (B01=1 OR B02=1 OR B03=1) and aware of Lyme disease (C01=2)**

C26 [Rating slider, show all statements on same screen]

Please rate your agreement with the following statements:

1. I’m confident I can avoid exposure to ticks with basic measures like bug spray and long pants.
2. I’m confident my child can avoid exposure to ticks with basic measures like bug spray and long pants
3. Even if I am in areas with a high risk of Lyme borreliosis, I can avoid contracting Lyme borreliosis by being careful
4. Even if my child is in areas with a high risk of Lyme borreliosis, I can avoid contracting Lyme borreliosis by being careful

Please use the scale from 1 to 5, where 1 is “Disagree strongly” and 5 is “Agree strongly”.


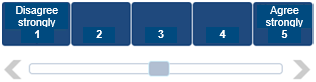


*SCRIPTER: Randomize statements*

**Section introduction: Outdoor activity**

**Base: All**

We will now ask a number of questions about outdoor activities commonly done between 1^st^ April and 30^th^ November. By “**outdoor activities**” we mean activities (e.g. walking, hiking, gardening, work, recreation, sports) that take place in forests, woods, parks, tall grass, or outdoors on your property.

For activities you never do, please indicate “0 hours” .

**Occupational activity**

**Base: All**

D01 [Q]

As a part of your primary occupation(s) (work, school, or volunteering), how many hours do you spend per week doing outdoor activities between 1^st^ April and 30^th^ November? If such occupations do not apply to you, please indicate “0” hours.

Approximately [….] hours per week

**Outdoors at home**

**Base: All**

D02 [Q, per activity]

On average, how many hours do you spend per week in your garden, or outdoors on your property between 1^st^ April and 30^th^ November?

    Outdoor activity at home

1. Gardening [….] hours per week
2. Mowing the lawn [….] hours per week
3. Reading/sunbathing [….] hours per week
4. Other outdoor activity at home [….] hours per week

[∑autosum] hours per week

*SCRIPTER: Randomize answers 1-3*

**Outdoors away from home**

**Base: All**

D03 [Q, per activity]

On average, how many hours do you spend doing the below outdoor activities away from home between 1^st^ April and 30^th^ November?

    Outdoor activity away from home

1. Bird watching [….] hours per week
2. Hiking/walking/running/biking [….] hours per week
3. Picnicking/grilling/eating outdoors [….] hours per week
4. Walking your dog [….] hours per week
5. Other outdoor activity away from home [….] hours per week

[∑autosum] hours per week

*SCRIPTER: Randomize answers 1-4*

**Outdoor trips**

**Base: All**

D04 [Q, per activity] and D05 [Q, per activity]

Between 1^st^ April and 30^th^ November, how often and for how long do you generally do the following outdoor activities in areas with forests, woods, parks, or tall grasses? Use “0” to indicate never.

|  |  | *[D04]* | *[D05]* |
| --- | --- | --- | --- |
| 1. | Fishing | […] times per year | […] **hours** per trip |
| 2. | Hunting | […] times per year | […] **hours** per trip |
| 3. | Camping / Wilderness backpacking | […] times per year | […] **days** per trip |
| 4. | Spending time outdoors, away from regular home (e.g. cabin, hotel etc.) | […] times per year | […] **days** per trip |

*SCRIPTER: Randomize statements, but group 1-2 and 3-4*

*SCRIPTER: Show D05 only if D04>0*

*SCRIPTER: Hidden variable: ∑Total hours/year. 3-4: convert days to hours by key 1 day = 16 hours.*

**Outdoor activity, child**

**Base: All with 1+ child (A03>0)**

D06 [Q]

Among the children under the age of 18 years living in your household (at least part time), please now think of the oldest. How many hours do they spend per week doing outdoor activities between 1^st^ April and 30^th^ November? If such occupations does not apply, please indicate “0” hours.

Approximately [….] hours per week

**Urbanity**

**Base: All**

E02 [S]

Which of the following best describes where you live?

1. I live in a city
2. I live in the suburbs of a city/ outlying residential district of a city
3. I live in the countryside

**Lived in area type X years**

**Base: All**

E02b [S]

How long have you lived in that area type (city/suburb/countryside)?

1. Up to 2 years
2. More than 2, less than 5 years
3. More than 5 years

**Having a dog**

**Base: All**

E03 [S]

Does your household have a dog?

1. Yes
2. No

**Removing ticks from dog**

**Base: All tick aware (B01=1 OR B02=1 OR B03=1) and has a dog (E03=1)**

E04 [Rating grid slider]

How often do you check your dog for ticks after a walk.

Please use the scale from “Always” to “Never”.

*SCRIPTER: Rating slider: Always/Often/Sometimes/Seldom/Never/I don´t know*

**Tick bite, pet**

**Base: All tick aware (B01=1 OR B02=1 OR B03=1) and has a dog (E03=1)**

E05 [Q]

During the last year, how many times was your household’s dog bitten by a tick, if at all? In case of several dogs, please think of the dog you have had for the longest.

____times during the last year

**..**

**Educational background**

**Base: All**

E06 [S]

Which one of the following best describes your educational background?

1. Approx. 9/10 years of school (e.g. elementary/primary school)

2. Approx. 11-13 years of school (e.g. high school/secondary school)

3. Approx. 14 or more years in total (e.g. university)

**Income**

**Base: All**

E07 [S]

What best describes the approximate monthly net income of your household?

1. < 250 €
2. 251-500 €
3. 501-750 €
4. 751-1.000 €
5. 1.001-2.000 €
6. 2.001-3.000 €
7. 3.001-4.000 €
8. 4.001-5.000 €
9. 5.001-6.000 €
10. 6.001-7.000 €
11. >7.001 €
12. I don´t know

*SCRIPTER: For non-EURO markets, see local questionnaire for equivalent brackets in local currency*

**Breaker**

Thank you for your participation so far, the survey is almost complete. We would just like to ask you 3 more questions on your relationship with your doctor and vaccinations in general.

**HCP trust**

**Base: All**

F00 [Rating slider, show all statements on same screen]

Please rate your agreement with the following statements:

1. I trust my doctor so much I always try to follow his/her advice
2. I sometimes distrust my doctor´s opinion and would like a second one
3. I trust my doctor to put my medical needs above all other considerations when treating my medical problems
4. My doctor is a real expert in taking care of medical problems like mine

Please use the scale from 1 to 5, where 1 is “Disagree strongly” and 5 is “Agree strongly”.


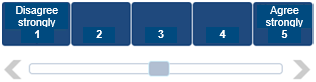


*SCRIPTER: Randomize statements*

*SCRIPTER: Provide also a “I don’t know” for each statement*

**Vaccine Attitude**

**Base: All**

F01 [Rating slider, show all statements on same screen]

Please rate your agreement with the following statements about vaccines in general:

1. In general, I feel safe after being vaccinated
2. In general, I feel protected after getting vaccinated
3. Vaccination programs in general are a big conspiracy
4. In general, authorities promote vaccination for financial gain, not for people´s health
5. In general, the risks from a vaccine are greater than the risks of the disease

Please use the scale from 1 to 5, where 1 is “Disagree strongly” and 5 is “Agree strongly”.


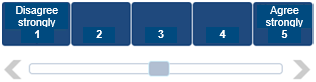


*SCRIPTER: Randomize statements*

*SCRIPTER: Provide also a “Prefer not to answer” for each statement*

*SCRIPTER: Do not show Code 3 and Code 4 for NO*

**Habit**

**Base: All**

F02 [Rating slider, show all statements on same screen]

Please rate your agreement with the following statements:

1. I normally receive all vaccines recommended by my health care provider
2. Accepting recommended vaccines is something I do automatically
3. I know exactly which doctor or pharmacy to go to get vaccines

Please use the scale from 1 to 5, where 1 is “Disagree strongly” and 5 is “Agree strongly”.


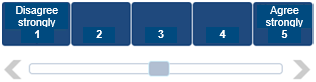


*SCRIPTER: Randomize statements*

*SCRIPTER: Provide also a “Prefer not to answer”*

**SCREENOUT TEXT**

*Over-quota screen-out text*

We’re sorry, but we have already reached a sufficient number of respondents matching your profile. Your opinion is important to us and we appreciate your time and continued participation in online surveys. Thank you.

*Closing text*

This was the end of the questionnaire. Thank you for your participation.

As mentioned in the beginning, Ipsos GmbH, a global market research company, will be processing the data and providing the findings to a pharmaceutical company.

**END OF QUESTIONNAIRE**

**Table S2.** Assessment of variation inflation factors (VIF) of independent predictor variables

| **Predictor** | **VIF Value** |
| --- | --- |
| Suburban | 5.16 |
| Rural | 5.25 |
| Middle-income status | 3.42 |
| Low-income status | 3.37 |
| 18–29 years | 1.27 |
| 30–39 years | 1.29 |
| 40–49 years | 1.28 |
| Female | 1.03 |
| Has dog | 1.08 |
| Tick bite history | 1.15 |
| Past LB diagnosis of self | 1.89 |
| Past LB diagnosis of friend/family | 1.15 |

**Table S3.** Crude odds ratio estimates evaluating predictor variables for Lyme borreliosis (LB) risk perception

| **Predictor** | **Crude OR (95% CI)** | **P-value** |
| --- | --- | --- |
| **Income level** | | |
| High (reference) | | |
| Middle income | 0.91 (0.84–0.99) | 0.03 |
| Low income | 0. 78 (0.71–0.87) | **<.001**** |
| **Urbanicity** | | |
| Urban (reference) | | |
| Suburban | 0.70 (0.65–0.76) | **<.001**** |
| Rural | 0.95 (0.88–1.03) | 0.23 |
| **Age** | | |
| 50–59 (reference) |  |  |
| 18–29 | 1.22 (1.11–1.34) | **<.001**** |
| 30–39 | 1.72 (1.57–1.88) | **<.001**** |
| 40–49 | 1.41 (1.30–1.53) | **<.001**** |
| **Gender** | | |
| Female (reference) |  |  |
| Male | 0.81 (0.76–0.87) | **<.001**** |
| **Owns a household dog** | 1.82 (1.70–1.95) | **<.001**** |
| **Ever had a tick bite** | 2.43 (2.27–2.60) | **<.001**** |
| **LB diagnosis history** |  |  |
| Ever personally had an LB diagnosis | 7.03 (6.25–7.90) | **<.001**** |
| Knows someone who ever had an LB diagnosis | 2.95 (2.76–3.16) | **<.001**** |

**Table S4.** Outdoor activity engagement by income status and urbanicity; counts and proportions have been weighted by country region, gender, and age*

|  | **Income status** | | | **Urbanicity** | | |
| --- | --- | --- | --- | --- | --- | --- |
| **Activity type** | **High income  n, N, %** | **Middle income n, N, %** | **Low income n, N, %** | **Urban  n, N, %** | **Suburban n, N, %** | **Rural n, N, %** |
| Any time reported hunting or fishing | 416, 3895, 10.7% | 672, 9680, 6.9% | 304, 3851, 7.9% | 792, 7793, 10.2% | 292, 4990, 5.9% | 308, 4644, 6.6% |
| Any time reported camping/wilderness backpacking | 3527, 4436, 79.5% | 7228, 10069, 71.8% | 2326, 3943, 59.0% | 6326, 8496, 74.5% | 3485, 5184, 67.2% | 3271, 4770, 68.6% |
| Any time outdoors away from regular home in forested areas | 3637, 4472, 81.3% | 7772, 10423, 74.6% | 2485, 4020, 61.8% | 6787, 8792, 77.2% | 3662, 5279, 69.4% | 3445, 4844, 71.1% |

*Results do not include upper-bound outliers, defined as: *Third quartile + 1.5 * Interquartile range*
